# Plant Root Exudates Are Involved in *Bacillus cereus* AR156 Mediated Biocontrol Against *Ralstonia solanacearum*

**DOI:** 10.3389/fmicb.2019.00098

**Published:** 2019-01-31

**Authors:** Ning Wang, Luyao Wang, Kai Zhu, Sensen Hou, Lin Chen, Dandan Mi, Ying Gui, Yijun Qi, Chunhao Jiang, Jian-Hua Guo

**Affiliations:** ^1^Department of Plant Pathology, College of Plant Protection, Nanjing Agricultural University, Nanjing, China; ^2^Jiangsu Collaborative Innovation Center of Regional Modern Agriculture & Environmental Protection, Nanjing, China; ^3^Key Laboratory of Integrated Management of Crop Diseases and Pests, Ministry of Education, Nanjing Agricultural University, Nanjing, China; ^4^Tsinghua-Peking Center for Life Sciences, Beijing, China; ^5^Center for Plant Biology, School of Life Sciences, Tsinghua University, Beijing, China

**Keywords:** biocontrol, plant root exudates, GC-MS, biofilm, tomato bacterial wilt

## Abstract

The biological control process mediated by microbes relies on multiple interactions among plants, pathogens and biocontrol agents (BCAs). One such efficient BCA is *Bacillus cereus* AR156, a bacterial strain that controls a broad spectrum of plant diseases and potentially works as a microbe elicitor of plant immune reactions. It remains unclear, however, whether the interaction between plants and *B. cereus* AR156 may facilitate composition changes of plant root exudates and whether these changes directly affect the growth of both plant pathogens and *B. cereus* AR156 itself. Here, we addressed these questions by analyzing the influences of root exudate changes mediated by *B. cereus* AR156 during biocontrol against tomato bacterial wilt caused by *Ralstonia solanacearum.* Indeed, some upregulated metabolites in tomato root exudates induced by *B. cereus* AR156 (REB), such as lactic acid and hexanoic acid, induced the growth and motile ability of *in vitro B. cereus* AR156 cells. Exogenously applying hexanoic acid and lactic acid to tomato plants showed positive biocontrol efficacy (46.6 and 39.36%) against tomato bacterial wilt, compared with 51.02% by *B. cereus* AR156 itself. Furthermore, fructose, lactic acid, sucrose and threonine at specific concentrations stimulated the biofilm formation of *B. cereus* AR156 in Luria-Bertan- Glycerol- Magnesium medium (LBGM), and we also detected more colonized cells of *B. cereus* AR156 on the tomato root surface after adding these four compounds to the system. These observations suggest that the ability of *B. cereus* AR156 to induce some specific components in plant root exudates was probably involved in further biocontrol processes.

## Introduction

*Bacillus cereus* AR156 is a BCA strain with biocontrol efficacy against a broad spectrum of plant diseases, such as tomato bacterial wilt caused by *Ralstonia solanacearum* and tomato root-knot caused by *Meloidogyne incognita* (Wei et al., 2010; Niu et al., 2012). During the biocontrol process mediated by *B. cereus* AR156, the induction of defense-related genes, including *PR1, PR2*, and *PR5*, was observed in *Arabidopsis* plants (Niu et al., 2011). *B. cereus* AR156 was proven to be a microbe elicitor of plant defenses that triggers rhizobacteria-induced systemic resistance (ISR) in *Arabidopsis*, tomato plants and fruits in postharvest storage (Wang et al., 2013, 2014; Niu et al., 2016a,b; Nie et al., 2017). Although many studies have been performed to search for active substances with biocontrol efficacy within *B. cereus* AR156 or its metabolite products, little progress has been achieved, except for the finding of extracellular polysaccharides (EPS), known as one novel microbe-associated molecular patterns (MAMPs) (Jiang et al., 2016). According to our previous results, EPS of *B. cereus* AR156 elicited a strong hypersensitive response (HR), causing hydrogen peroxide accumulation, callose deposition and induction of defense-related enzyme activity in plants (Jiang et al., 2016).

Another biocontrol study of *B. cereus* AR156 considers its ability to influence the growth and production of host plants (Zhou et al., 2016). It is well documented that plant root exudates are important for mediating plant-microbe interactions in the rhizosphere (Narula et al., 2009). Different plants have different root exudate compositions, which are also regulated by the developmental stage of plants and numerous environmental factors, such as humidity, pH, and soil type (Dakora and Phillips, 2002; Bertin et al., 2003; Barak and Schroeder, 2012). Although not necessary, plant root exudates were reported to promote biofilm formation by beneficial bacteria on the root surface (Zhang et al., 2014).

Furthermore, ATP-binding cassette (ABC) transporters, which facilitate the transportation of compounds over the plasma membrane and into vacuoles (Yazaki, 2006), are involved in plant-microbe interactions by modulating plant root exudation (Hückelhoven, 2007). In a previous study, *B. cereus* AR156 promoted the shoot growth of *Arabidopsis* Col-0 and *Atabcg30*. However, *B. cereus* AR156 repressed the growth of *Atabcc5.* The root exudates of Col-0 induced the expression of siderophore and chitinase production-related genes in *B. cereus* AR156, but root exudates of *Atabcc5* showed contradictory effects on the expression level of those genes (Zhou et al., 2016). ABC transporters play key roles during interactions with rhizosphere organisms and are essential for plant root exudation (Loyola-Vargas et al., 2007; Sugiyama et al., 2007; Badri et al., 2008, 2009). These previous studies indicated the diversity of different compositions involved in plant root exudate-mediated interactions between plants and *B. cereus* AR156, and it would be interesting to study the potential role of *B. cereus* AR156 in reversely manipulating the components in plant root exudates.

A previous study by Liu et al. (2017) highlighted that root exudates were altered in cucumber plants preinoculated with the BCA strain *Bacillus amyloliquefaciens* SQR9 or the fungal pathogen *Fusarium oxysporum* f. sp. *cucumerinum*. Eight out of 109 compounds were significantly correlated with root colonization of either SQR9 or *F. oxysporum*, during which tryptophan strengthened further colonization of SQR9 while raffinose was reduced to inhibit root colonization of *F. oxysporum* f. sp. *cucumerinum* (Liu et al., 2017). As an effective broad-spectrum BCA strain, it is meaningful to understand whether *B. cereus* AR156 plays a similar role as that of SQR9 in controlling soilborne pathogens, such as *R. solanacearum.*

Plant root exudates contain a large group of components involved in such diverse processes as plant growth regulation, plant signaling and microbe-plant interactions (Narula et al., 2009). Based on GC-MS analysis, different compounds in root exudates were identified, such as sugars, amino acids, organic acids, fatty acids (Suzuki et al., 2009), sugar alcohols, phenolics (Chaparro et al., 2013), and phosphates (Bowsher et al., 2015). In this study, a split-root system similar to that used in Liu et al.’s work was used to divide tomato roots into two parts, and root exudate collection was spatially separated from *B. cereus* AR156 treatment to avoid interference of bacterial metabolites (Liu et al., 2017). We explored further biocontrol mechanisms of *B. cereus* AR156 by investigating composition changes in plant root exudates from tomato plants treated with *B. cereus* AR156 and whether these changes had a positive effect on biocontrol against tomato bacterial wilt caused by *R. solanacearum*. Subsequently, compounds detected in tomato root exudates induced by *B. cereus* AR156 treatment were further tested for their influences on *R. solanacearum* and *B. cereus* AR156, such as relative growth increment, swarming ability, biofilm formation ability and root colonizing ability.

## Materials and Methods

### Bacterial Strains, Plants, and Growth Conditions

*Bacillus cereus* AR156 was isolated from the forest soil of Zhenjiang City, Jiangsu Province, China, identified as an effective bacterial BCA (Genebank accession number CP015589, China General Microbiology Culture Collection Center, CGMCC accession number 1929), and grown in LB medium (NaCl 10 g⋅L^-1^, yeast extract 5 g⋅L^-1^, tryptone 5 g⋅L^-1^) at 30°C on agar plates or in liquid medium for 24 h (250 rpm). *R. solanacearum* HN4 was isolated from infected tomato plant samples in Hunan Province, China, and grown in YGPA medium (yeast extract 5 g⋅L^-1^, peptone 5 g⋅L^-1^, glucose 10 g⋅L^-1^) at 28°C on solid plates or in liquid media for 24 h (250 rpm).

Seeds of tomato (*Solanum lycopersicum*) Hezuo 903 (purchased from Research Institute of Vegetables, Academy of Agricultural Sciences, Jiangsu Province, China) were spread in seedling culture plates after surface sterilization. Tomato plants were transferred into independent pots at the 4-leaf stage and then moved to environment-controlled growth chambers under long-day conditions (16 h light/8 h dark cycle at 140 μE/s/m^2^ light intensity) at 22°C.

### Tomato Root Exudates Collection

**FIGURE 1:**
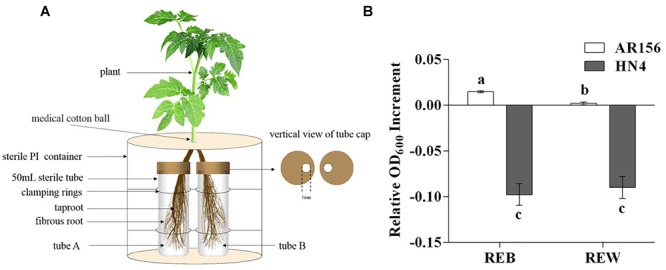
Tomato root exudates collection and effects on relative growth increment of *Bacillus cereus* AR156 and *Ralstonia solanacearum* HN4. **(A)** Tomato root exudates were collected in a split-root system. Root bundle in tube A was inoculated with water or cell culture of *B. cereus* AR156, and root exudates was collected in tube B with spatial separation from potential interference of *B. cereus* AR156 metabolite. **(B)** Relative growth increment of *Bacillus cereus* AR156 and *Ralstonia solanacearum* HN4 in minimal medium containing different root exudates. Tomato root exudate induced by *Bacillus cereus* AR156 (1 × 10^7^ CFU/mL) (REB); Tomato root exudate induced by sterile water (REW). The mean and standard error values of three biological replicates are reported for each treatment. Different letters within a column indicate significantly differences among treatments according to the Duncan’s multiple range tests test (*P <* 0.05). The experiment was carried out three times and one representative experiment is reported.

Tomato root exudates were collected using the following procedures. Tomato plants in the 4-leaf stage were isolated from soil (the soil used in this study was characterized as yellow brown earth with pH 5.8 (1:5), organic matter content of 23.8 g/kg, total nitrogen of 6.5 g/kg, available phosphorus of 184.3 mg/kg, and available potassium of 212.25 mg/kg; the soil texture was 184 g/kg of clay, 540 g/kg of silt, 276 g/kg of sand), gently washed with sterilized water three times, and then transferred into Hoagland’s nutrient solution for 3 days. The roots of each tomato plant were divided into two parts, and each part was immersed into a 50 mL centrifuge tube with 40 mL of sterilized water. Five milliliters of *B. cereus* AR156 culture (1 × 10^7^ CFU⋅ml^-1^) was added to one of the tubes for bacterial treatment (tube A) (control treatment was conducted with sterilized water), as described in Figure 1A. After 3 days, the solution in the other tube (tube B), which was considered to contain non-treated tomato root exudates only, was filtered by a 0.22 μm sterilized membrane and then freeze concentrated to 5 mL final volume. Each treatment contained six tomato plants, and each plant provided approximately 0.1 g of fresh roots. The weight of lyophilized exudates was 43.70 ± 2.93 mg in each treatment. Six independent repeats were conducted in this root exudate collection procedure.

### Analysis of Root Exudates

Collection of root exudates: 500 μL of thawed tomato root exudates and 2 mL of cold methanol including internal standard (250 ng/mL L-norleucine) were mixed and vortexed for 5 s. Following centrifugation at 16,000 *g* at 4°C for 15 min, 2.2 mL of supernatant was dried under a nitrogen stream. The residue was reconstituted in 30 μL of 20 mg/mL methoxyamine hydrochloride in pyridine, and the resulting mixture was incubated at 37°C for 90 min. A 30 μL aliquot of BSTFA (with 1% TMCS) was added to the mixture and derivatized at 70°C for 60 min prior to GC-MS metabolomics analysis. Quality control (QC) samples pooled from samples in each group were prepared and analyzed with the same procedure as that for the experimental samples.

Metabolite profiling of root exudates: Metabolite profiling was performed on an Agilent 7890A gas chromatography system coupled to an Agilent 5975C inert MSD system (Agilent Technologies Inc., Santa Clara, CA, United States). An Rxi-5 Sil MS fused-silica capillary column (30 m × 0.25 mm × 0.25 μm; Restek corporation, Bellefonte, PA, United States) was utilized to separate the derivatives. Helium (>99.999%) was used as a carrier gas at a constant flow rate of 1 mL/min through the column. The injection volume was 1 μL in splitless mode, and the solvent delay time was 6 min. The initial oven temperature was held at 70°C for 2 min; ramped to 160°C at a rate of 6°C/min, to 240°C at a rate of 10°C/min, and to 300°C at a rate of 20°C/min; and finally held at 300°C for 6 min. The temperatures of the injector, transfer line, and electron impact ion source were set to 250, 250, and 230°C, respectively. The impact energy was 70 eV, and data were collected in full scan mode (m/z 50–600).

Concentrations of each metabolite in REB or REW were calculated by comparison of analytes with standard sample as described by previous report (Pätzold et al., 2005).

### Bacterial Relative Growth Increment Assays

Assays of the bacterial relative growth increment followed a previously reported method with some modifications (Huang et al., 2017). Briefly, overnight fresh culture of *B. cereus* AR156 or *R. solanacearum* HN4 were centrifuged at 8000 rpm for 5 min and suspended in 0.85% NaCl solution to OD_600_ = 0.1. Five microliters of the bacterial solution was added to 150 μL of MM medium (minimum nutrient medium, L-asparagine 0.5 g⋅L^-1^, K_2_HPO_4_ 0.5 g⋅L^-1^, MgSO_4_⋅7H_2_O 0.2 g⋅L^-1^, FeSO_4_.7H_2_O 0.01 g⋅L^-1^, glucose 10 g⋅L^-1^) (Chen et al., 2012) in a 96-well plate. Tomato root exudate induced by *Bacillus cereus* AR156 (REB), or sterile water (REW), at final concentrations of 10, 1, 0.1, or 0.01 mM for each specific root exudate component (fructose, lactic acid, D-pinitol, sucrose, threonine, and hexanoic acid) was added to the 96-well plates, and 0.85% NaCl was considered the control treatment. The plates were then moved into a shaking incubator for 24 h (28°C, 200 rpm), and OD_600_ reads were detected with a SpectraMax M5 plate reader (Molecular Devices, Sunnyvale, CA, United States). The bacterial relative growth increment was represented by its OD_600_ increment and calculated by the following formulas:

OD600 Increment = OD600 read in treated sample - OD600 read in control sampleRelative OD600 Increment= 24 hpt* OD600 increment - 0 hpt OD600 increment(*hpt represents `hours post treatment′)

### Analysis of Bacterial Motility

A bacterial motile ability assay followed previous studies with few modifications (Wang et al., 2017; Wu et al., 2017). A total of 10, 1, 0.1, or 0.01 mM each root exudate component was added to 0.5% LB agar plates (for *B. cereus* AR156) and 0.7% YGPA plates (for *R. solanacearum* HN4) and air dried for 30 min for further bacterial motile ability assays. One milliliter of overnight culture of *B. cereus* AR156 or *R. solanacearum* HN4 (OD_600_ = 0.8) was centrifuged at 5000 rpm for 3 min, suspended in 1 mL of PBS buffer (KH_2_PO_4_ 0.27 g⋅L^-1^, Na_2_HPO_4_ 1.42 g⋅L^-1^, NaCl 8 g⋅L^-1^, KCl 0.2 g⋅L^-1^, pH = 7.4) twice, and then dissolved in 100 μL of PBS buffer. Five microliters of the bacterial PBS solution was dropped in the center of each prepared 0.5% LB agar plate and 0.7% YGPA plate and dried in a biosafety cabinet for 10 min. All plates were moved into a 28°C incubator and held for 10 h for *B. cereus* AR156 and 5 days for *R. solanacearum* HN4, and the diameter of bacterial colonies on each plate was measured. Relative bacterial motile ability was calculated by the following formula:

$$\begin{array}{c} Relative bacterial motile ability = diameter of bacterial colony on treatment plate-diameter of bacterial colony on control plate \end{array}$$

### Antagonism Ability Assay

An antagonistic ability assay was carried out as previously described with few modifications (Xue et al., 2009). One milliliter of fresh culture of *R. solanacearum* HN4 (1 × 10^7^ CFU⋅ml^-1^, OD_600_ = 0.5) was mixed with 50 mL of melted YGPA agar medium and poured into an 8 cm petri dish with a grid pattern. Five microliters of *B. cereus* AR156 culture (1 × 10^8^ CFU⋅ml^-1^, OD_600_ = 1), supernatant of *B. cereus* AR156 culture or specific root exudate components in selected concentrations was dropped onto 5 mm sterilized filter paper disks. A culture solution of *Bacillus amyloliquefaciens* HSSN09 strain (a previously isolated BCA strain with broad-spectrum antagonistic ability against fungal and bacterial pathogens, China General Microbiology Culture Collection Center, CGMCC accession number 15764) and its supernatant were considered positive controls in this assay. The filter paper disks were air dried for 20 min and then placed in prepared petri dishes. All petri dishes were placed upside down in a 28°C incubator for 48 h, and the antagonistic area for each treatment was recorded. The inhibition zone radius was calculated using the following formula:

$$\begin{array}{c} Inhibition zone radius = radius of transparent inhibition zone- radius of bacterial colony (or sterilized filter paper disks) in antagonism plate \end{array}$$

### Biocontrol Assay

To test the efficacy of BCA strains or specific root exudate components, we followed the method described previously with few modifications (Chen et al., 2013). Tomato seeds were surface-sterilized and germinated on moist potting soil. Seeds were sown into soil (same type as described above) and cultivated in a growth chamber (16/8 h light/dark photoperiod). After approximately 2 weeks, tomato plants were transferred into pots (8 cm × 9 cm × 12 cm) filled with soil fertilized with a fertilizer having the rate of N-P-K, 22-10-13. Twenty milliliters of *B. cereus* AR156 cell culture, adjusted to a cell density of 1 × 10^8^ CFU⋅ml^-1^ by resuspension in 0.85% NaCl solution, or 10 mM each specific root exudate component (lactic acid and hexanoic acid) was applied as irrigation to each pot. One week after inoculation with BCAs or specific root exudates, 20 mL of *R. solanacearum* HN4 cell suspension (1 × 10^7^ CFU⋅ml^-1^) was drenched into each pot. The temperature in the growth chamber was then maintained at 28°C, the relative humidity was maintained at 30%, and the photoperiod was 16 h/8 h.

Thirty days after inoculation with the pathogen, disease incidence based on the disease index (DI) was recorded as follows: DI = 0, no wilt; DI = 1, wilt symptoms on 1–25% of the leaves; DI = 2, wilt symptoms on 26–50% of the leaves; DI = 3, wilt symptoms on 51–75% of the leaves; and DI = 4, wilt symptoms on more than 76% of the leaves. Disease incidence and biocontrol efficacy were calculated according to the following formula:

$$\begin{array}{c} Disease severity(\%) = [\sum (Number of diseased plants in this index \times disease index)/(Total number of plants investigated \times highest disease index)] \times 100.\\ Biocontrol efficacy(\%) = [(Disease severity of control plants -Disease severity of treated plants) /Disease severity of control] \times 100. \end{array}$$

Each treatment had three replicate, and each replica contained 24 tomato plants. The experiment had three biological repeats.

### *In vitro* Biofilm Formation Assay

The *in vitro* biofilm formation assay of *B. cereus* AR156 followed a previously reported method with few modifications (Yan et al., 2017). Four microliters of overnight-grown bacterial culture (1 × 10^8^ CFU⋅ml^-1^, OD_600_ = 1) was added to 12-well plates with 4 mL of newly formulated LBGM medium (Shemesh and Chai, 2013), and each well contained one of the root exudate components at different concentrations. The plates were placed at 30°C for 2 days without movement, and images were then taken by a Cannon DS126311 camera.

For quantitative analysis of biofilm formation, 10 μL of overnight-grown bacterial culture was added to 96-well microliter plates with 100 μL of LBGM medium and root exudate components at specific concentrations. The plates were then placed at 30°C for 2 days. Contents of the microliter plate were poured off and washed with 300 μL of PBS buffer three times. The remaining contents in each well were fixed with 250 μL of pure methanol for 15 min, and the plates were emptied and air dried. Crystal violet solution (1%) was then used to stain the biofilm structure in the wells for 5 min, and the excess stain was rinsed off with tap water. Glacial acetic acid (33%) was used to extract the dye bound to the biofilm structure, and the results were visualized with a SpectraMax M5 plate reader (Molecular Devices, Sunnyvale, CA, United States) at 570 nm.

### Root Colonization Assay

A wild-type strain of *B. cereus* AR156 that harbored a constitutively expressed gfp reporter plasmid, pGFP78 (Gao et al., 2015), was tested for its colonizing ability on tomato root surfaces. This assay followed previously reported methods by Chen et al. (2013) with few modifications. Surface-sterilized tomato seeds were transferred onto 0.7% MS agar plates and incubated at 25°C for 4 days to obtain tomato roots with lengths of at least 3 cm. Tomato roots were then transferred into 12-well microliter plates with each well containing 4 mL of MS medium, and the plates were placed in a shaker for 2 days (25°C, 60 rpm). A cell suspension of overnight-grown *B. cereus* AR156 harboring pGFP78 was then added to root-MS medium to reach a final OD_600_ = 0.1, and different root exudate components were added to this system to reach specific concentrations as well. After 3 days of incubation at 25°C, all the roots were carefully collected and observed by CLSM (Leica AF6000 modular microsystems) according to a previously reported method (Chen et al., 2013).

### Statistical Analyses

One-way analysis of variance (ANOVA) was carried out with SPSS (Version 19.0) and followed with Duncan’s multiple range tests (*P < 0.05*) for statistically analyzed and Student’s *t*-test for evaluating the significance in all data. Peak picking, alignment, deconvolution, and further processing methods of raw GC-MS data were described in previously published protocols (Jin et al., 2014). The final data were exported as a peak table file, including observations (sample name), variables (rt_mz), and peak abundances (Supplementary Table S1). The data were normalized by Excel 2010 software against total peak abundances before performing univariate and imported into a SIMCA-P (version 13.0, Umetrics AB, Umea, Sweden). Principal component analysis (PCA) and orthogonal partial least-squares discriminant analysis (OPLS-DA) were then processed.

The differential metabolites were acquired based on the consideration of a statistically significant threshold of variable influence on projection (VIP) values obtained from the OPLS-DA model and *p*-values from a two-tailed Student’s *t*-test on the normalized peak areas, in which metabolites with VIP values larger than 1.0 and *p-*values less than 0.05 were included, respectively. Fold change was calculated as a binary logarithm of the average normalized peak area ratio between group REB and REW.

## Results

### Effect of Plant Root Exudates Mediated by *B. cereus* AR156 on Growth of *B. cereus* AR156 and *R. solanacearum* HN4

Figure 1A displays the exact method that this study used for tomato root exudate collection, and Figure 1B shows changes in the relative OD_600_ value in *B. cereus* AR156 and *R. solanacearum* HN4 cell culture resulting from adding REB and REW. These results suggest that relative growth increment of *B. cereus* AR156 was promoted by 85.9 (±11.3) % in REB than in REW, but had no significant effects on *R. solanacearum* HN4 (Figure 1B).

### *B. cereus* AR156 Alters Compositions of Tomato Root Exudates

**FIGURE 2:**
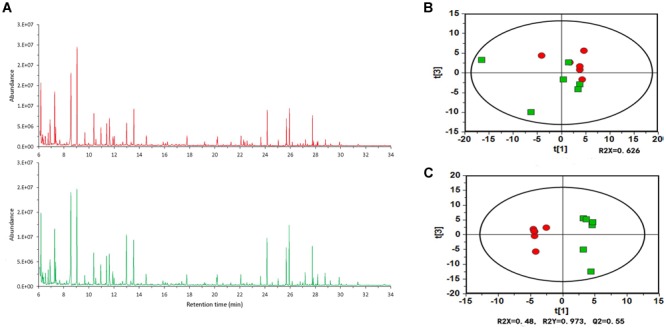
GC-MS TIC chromatograms **(A)**, principal component analysis (PCA) **(B)** and orthogonal partial least-squares discrimination analysis (OPLS-DA) scores plots **(C)** of tomato root exudates induced by *Bacillus cereus* AR156 (red) and sterile water (green).

To better understand the specific alterations in plant root exudates from tomato plants treated with *B. cereus* AR156, we performed a GC-MS analysis on tomato root exudates. Figure 2A shows the typical total ion current (TIC) chromatograms, and the peak abundance has a significant difference. To demonstrate the further differences between *B. cereus* AR156-treated and non-AR156-treated tomato root exudates, we utilized principal component analysis (PCA) and orthogonal partial least-squares discrimination analysis (OPLS-DA) using SIMCA13.0. Indeed, PCA and OPLS-DA revealed that the compounds detected in the *B. cereus* AR156- and non-AR156-treated root exudates analyzed by GC-MS were different from each other (Figure 2B,C). GC-MS analyses identified 141 features within *B. cereus* AR156-treated and non-AR156 treated root exudates, of which 75 features were annotated in Supplementary Table S1. Furthermore, it was observed that *B. cereus* AR156 induced the secretion of D-pinitol, fructose, threonine, lactic acid, sucrose, and hexanoic acid in tomato root exudates; however, the secretion levels of xylitol, myo-inositol, gamma-aminobutyric acid, and gluconic acid were suppressed in tomato root exudates treated with *B. cereus* AR156 compared with control root exudates according to FC > 0 and VIP > 1, *p* < 0.05.

### Antagonistic Ability of Tomato Root Exudate Components, Induced by *B. cereus* AR156, Against *R. solanacearum* HN4

**FIGURE 3:**
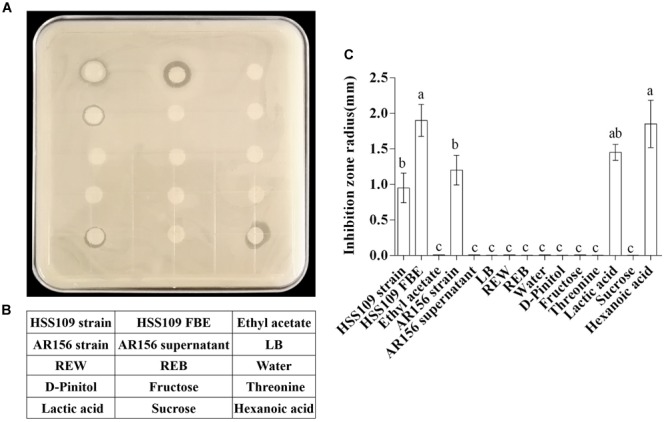
Antagonistic effects of components in tomato root exudates induced by *Bacillus cereus* AR156 against *Ralstonia solanacearum* HN4. **(A)** *In vitro* antagonism test of components in tomato root exudates induced by *B. cereus* AR156 against *R. solanacearum* HN4. Arrangement of tested compositions was described in **(B)**. *Bacillus amyloliquefaciens* HSSN09 and *Bacillus cereus* AR156 strains were tested in 5 × 10^7^ CFU/mL, HSSN09 FBE indicates the fermentation broth extract of *Bacillus amyloliquefaciens* HSSN09 in 20 mg/mL, tested root exudates was in the concentration of 8.74 mg/mL, other tested components were in the concentration of 100 mM. The results in **(A)** are quantified in **(C)**. The mean and standard error values of three biological replicates are reported for each treatment. Different letters within a column indicate significantly different among treatments according to the Duncan’s multiple range tests test (*P <* 0.05). The experiment was carried out three times and one representative experiment is reported.

Table 1 lists six major metabolites found in tomato root exudates from tomato plants treated with *B. cereus* AR156, including fructose, lactic acid, D-pinitol, sucrose, threonine, and hexanoic acid. Meanwhile, concentrations of these six major metabolites in crude extracts were 0.31, 0.72, 0.06, 0.17, 0.06, and 0.12 mM in REB, and were 0.15, 0.46, 0.04, 0.87, 0.03, and 0.10 mM in REW (Supplementary Figure S1). *In vitro* antagonism tests were then conducted between *R. solanacearum* HN4 and these 6 metabolites in different concentrations. In Supplementary Figure S2, there were no antagonism effect detected in 0.01, 0.1, 1, 10, 50, and 100 mM fructose, D-pinitol, sucrose, and threonine. Interestingly, 50 and 100 mM lactic acid and hexanoic acid showed antagonism abilities against *R. solanacearum* HN4 (Supplementary Figure S2 and Figure 3), but in low concentrations performed no antagonism effects (Supplementary Figure S2). The *B. amyloliquefaciens* HSSN09 culture (5 × 10^7^ CFU⋅ml^-1^) and its fermentation broth extract (20 mg/mL) also displayed antagonistic activity against *R. solanacearum* HN4 and were used as a positive control (Figure 3A). The results in Figure 3A were quantified in Figure 3C by calculating the inhibition zone radius. *B. cereus* AR156 cultures (5 × 10^7^ CFU⋅ml^-1^) showed antagonism against *R. solanacearum* HN4, while its cell culture supernatant had no such effect in the same experiment. Meanwhile, crude root extracts induced by *B. cereus* AR156 showed no *in vitro* antagonism effect against *R. solanacearum* HN4 (REB, Figure 3), which was likely caused by low concentrations of key metabolites in crude root extracts.

**Table 1:** Components in tomato root exudates induced by *B. cereus* AR156.

Metabolites	VIP^a^	*P*-value^b^	FC (REB/REW)^c^	KEGG^d^	Related pathway
Fructose	1.68	0.045	1.17	C02336	Amino sugar and nucleotide sugar metabolism
Lactic acid	1.77	0.032	0.69	C00186	Glycolysis/ Gluconeogenesis; Fructose and mannose metabolism; Pyruvate metabolism
D-pinitol	1.68	0.044	0.52	C03844	—
Sucrose	1.86	0.022	1.12	C00089	Galactose metabolism; Starch and sucrose metabolism
Threonine	1.71	0.040	0.87	C00188	Glycine, serine and threonine metabolism; Valine, leucine and isoleucine biosynthesis
Hexanoic acid	2.04	0.009	0.39	C01585	Fatty acid

*^*a*^VIP values, Variable Importance in the Projection, was obtained from the OPLS-DA model. ^*b*^*P*-value was calculated from a two-tailed Student’s *t*-test. ^*c*^FC (REB/REW), fold change, was calculated as a binary logarithm of the average mass response (normalized peak area) ratio between Group REB vs. Group REW, where a positive value means that the average mass response of the metabolite in Group REB is larger than that in Group REW. ^*d*^KEGG: Kyoto Encyclopedia of Genes and Genomes http://www.kegg.jp/.*

### Tomato Root Exudate Components Influence the Growth and Motile Ability of *B. cereus* AR156

**FIGURE 4:**
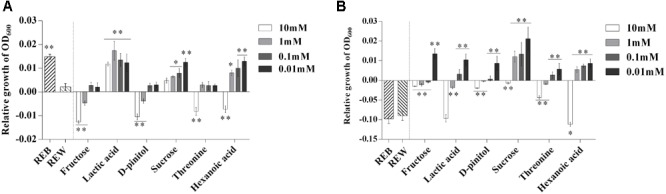
Relative growth increment of *Bacillus cereus* AR156 **(A)** and *Ralstonia solanacearum* HN4 **(B)** in minimal medium (MM) containing tomato root exudate induced by *Bacillus cereus* AR156 (REB), tomato root exudate induced by water (REW) or different components from tomato root exudates induced by *B. cereus* AR156 at final concentrations of 0.01, 0.1, 1, and 10 mM. The mean and standard error values of three biological replicates are reported for each treatment. Asterisks in **(A,B)** indicate significance difference between data of treatments and data of tomato root exudate induced by sterile water (REW) within each experiment as determined by the Student’s *t*-test (^∗^*P <* 0.05, ^∗∗^*P <* 0.01). The experiment was carried out three times and one representative experiment is reported.

To further explore the role of the *B. cereus* AR156-induced components in tomato root exudates, we performed a relative growth increment assay of both *B. cereus* AR156 and *R. solanacearum* HN4 in the presence of each induced root exudate component (Figure 4). Figure 4A shows that 10 mM, 1 mM fructose and D-pinitol, and 10 mM threonine and hexanoic acid clearly decreased the relative growth increment of *B. cereus* AR156. Moreover, lactic acid in all tested concentrations (10, 1, 0.1, and 0.01 mM), 1, 0.1, and 0.01 mM sucrose and hexanoic acid, as well as REB, caused growth increment of *B. cereus* AR156 compared with REW treatment (Figure 4A).

Unlike their effects on the growth increment of *B. cereus* AR156, only 10 mM hexanoic acid showed significant downregulation to the relative growth increment of *R. solanacearum* HN4 compared with REW (Figure 4B).

**FIGURE 5:**
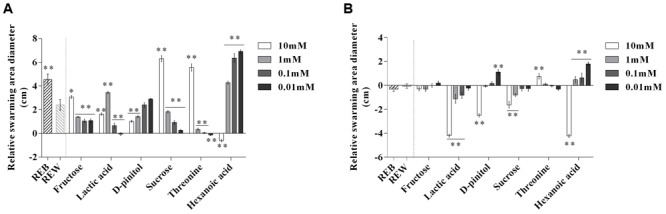
Relative swarming motility of *Bacillus cereus* AR156 **(A)** and *Ralstonia solanacearum* HN4 **(B)** on 0.5% Luria-Bertan (LB) or 0.7% Yeast-Glucose-Peptone-Agar (YGPA) plates amended with tomato root exudate induced by *Bacillus cereus* AR156 (REB), tomato root exudate induced by water (REW) or different components from tomato root exudates induced by *B. cereus* AR156 at final concentrations of 0.01, 0.1, 1, and 10 mM centrally inoculated with 5 μl of AR156/HN4 cells freshly grown (OD = 0.8) and incubated for 10 h and 5 days respectively at 28°C. The mean and standard error values of six biological replicates are reported for each treatment. Asterisks in **(A,B)** indicate statistical significance difference between data of treatments and data of tomato root exudate induced by sterile water (REW) within each experiment as determined by the Student’s *t*-test (^∗^*P <* 0.05, ^∗∗^*P <* 0.01). The experiment was carried out three times and one representative experiment is reported.

Next, we investigated whether these components in REB, can interfere with the motile ability of *B. cereus* AR156 and *R. solanacearum* HN4. The results showed 1 mM lactic acid, 10 mM sucrose, 10 mM threonine, 1, 0.1, and 0.01 mM hexanoic acid showed significant effects on motile ability of *B. cereus* AR156 (Figure 5A). Actually, REB treatment also showed positive effects on the motile ability of *B. cereus* AR156 (Figure 5A). Figure 5B shows that 10, 1, 0.1 mM lactic acid, 10 mM D-pinitol, 10 mM, 1 mM sucrose and 10 mM hexanoic acid significantly reduced the motile ability of *R. solanacearum* HN4 compared with REW, but REB had no such effect.

### Lactic Acid and Hexanoic Acid From Tomato Root Exudates Have Positive Biocontrol Efficacy Against Tomato Bacterial Wilt

**FIGURE 6:**
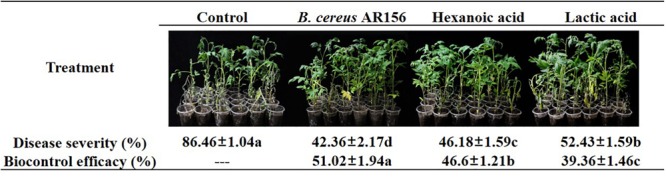
Biocontrol efficacy of *Bacillus cereus* AR156 and different components from tomato root exudates induced by *B. cereus* AR156 (Hexanoic acid and Lactic acid) against bacterial wilt caused by *Ralstonia solanacearum* HN4 under greenhouse condition. The mean and standard error values of three biological replicates are reported for each treatment. Different letters within a row indicate significantly differences among treatments by the Duncan’s multiple range tests test (*P <* 0.05). The experiment was carried out three times and one representative experiment is reported.

The initial goal of this study was to investigate whether the variation in plant root exudates from tomato plants treated with *B. cereus* AR156 had a positive function during the biocontrol process. In a biocontrol assay against tomato bacterial wilt, 10 mM hexanoic acid decreased the disease severity from 86.46 to 46.18%, and the biocontrol efficacy was 46.6%; 10 mM lactic acid decreased the disease severity from 86.46 to 52.43%, and the biocontrol efficacy was 39.36% (Figure 6), although both hexanoic acid and lactic acid showed lower biocontrol efficacy compared with *B. cereus* AR156 (51.02%) in the same assay.

### Tomato Root Exudate Components Affect Biofilm Formation and Root Colonizing Ability of *B. cereus* AR156

**FIGURE 7:**
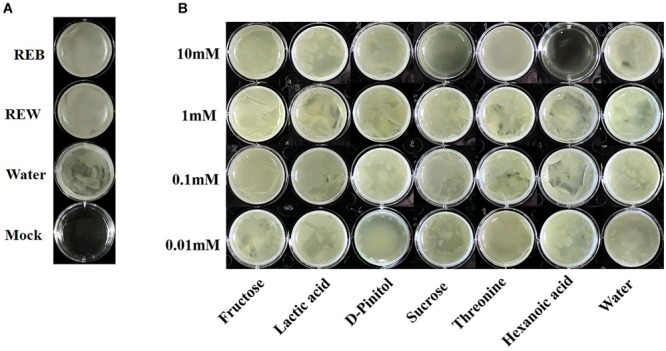
Pellicle biofilm formation of *Bacillus cereus* AR156 in Luria-Bertan- Glycerol- Magnesium medium (LBGM) containing tomato root exudate induced by *Bacillus cereus* AR156 (REB), tomato root exudate induced by water (REW) **(A)** or different components from tomato root exudates induced by *B. cereus* AR156 **(B)**. The experiment was carried out three times and one representative experiment is reported.

**FIGURE 8:**
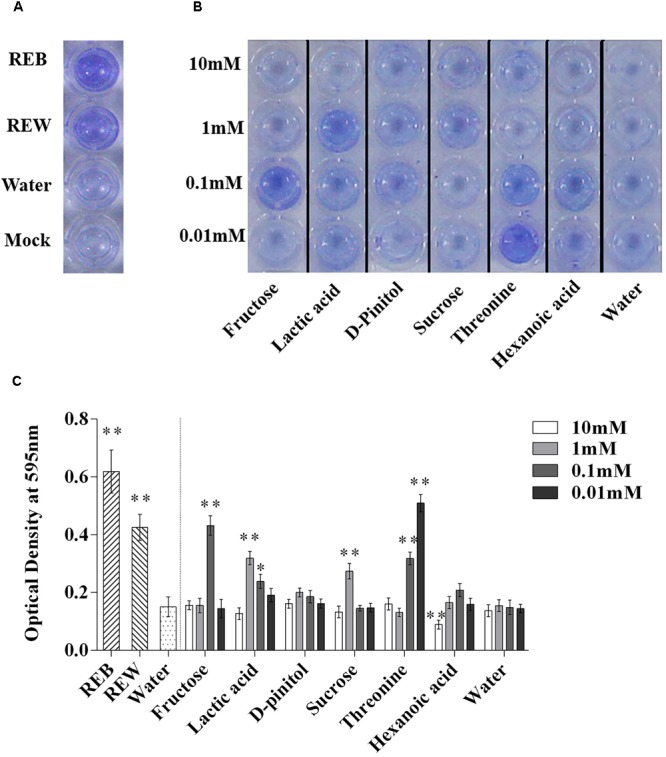
Biofilm formation on polystyrene microtiter plates by *Bacillus cereus* AR156 with still at 30°C for 48 h under microaerobic conditions. Biofilm formation were determined by crystal violetin in the 96-well microtiter plate induced by *Bacillus cereus* AR156 (REB), tomato root exudate induced by water (REW) **(A)** or different components from tomato root exudates induced by *B. cereus* AR156 **(B)**. **(C)** Relative optical density of *B. cereus* AR156’s biofilm was measured at 595 nm with a microtiter plate reader. The mean and standard error values of three biological replicates are reported for each treatment. Asterisks in **(C)** indicate statistical significance difference between data of treatments and data of sterile water as determined by the Student’s *t*-test (^∗^*P <* 0.05, ^∗∗^*P <* 0.01). The experiment was carried out three times and one representative experiment is reported.

*Bacillus cereus* AR156 was able to form a biofilm pellicle on the air-medium interface, and *B. cereus* AR156 formed a more robust or frailer biofilm structure when treated with different tomato root exudate components (Figure 7B). Crystal violet staining was conducted to quantify the biofilm formation capacity of *B. cereus* AR156 in different treatments. In Figure 8B,C, 0.1 mM fructose, 1 mM lactic acid, 1 mM sucrose, and 0.1 mM and 0.01 mM threonine significantly induced the biofilm formation capacity of *B. cereus* AR156, which was consistent with the results shown in Figure 7B. Specifically, all root exudate components in the highest tested concentration (10 mM) had no positive effects on the biofilm formation of *B. cereus* AR156, and 10 mM hexanoic acid impaired the biofilm formation, shown in both Figure 7, 8. In addition, both REB and REW stimulated the biofilm forming of *B. cereus* AR156 in LBGM medium, and the REB induced most robust biofilm formation of *B. cereus* AR156 compared with other specific components in all tested concentrations (Figure 7, 8).

**FIGURE 9:**
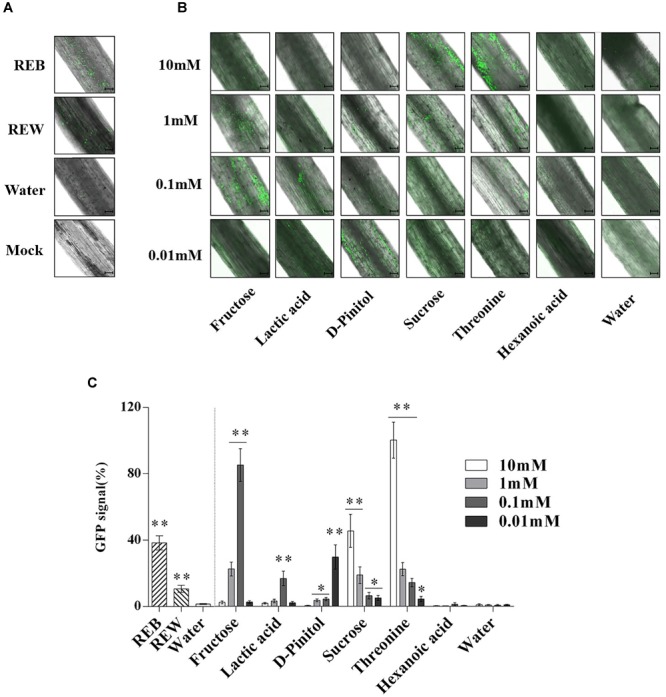
Attachment of GFP-targeted *Bacillus cereus* AR156 strain on tomato root surface. Cells of *Bacillus cereus* AR156 form different colonization on the surfaces of excised tomato roots in 1% MS liquid medium containing tomato root exudate induced by *Bacillus cereus* AR156 (REB), tomato root exudate induced by water (REW) **(A)** or different components from tomato root exudates induced by *B. cereus* AR156 **(B)** at 25°C for 3 days and were visualized by Confocal Laser Scanning Microscope. Mock showed tomato root grown in absence of *B. cereus* AR156 cells. Scale bars = 100 μm. The results in **(A,B)** were quantified in **(C)**. GFP signal was quantified using the Confocal Laser Scanning Microscope software (Leica AF6000 Modular microsystems) by measuring the total GFP fluorescence in one field inside the infiltration area with a low magnification objective (20X); all images used for fluorescence measurement were taken with the same settings. Basal signal measured in mock was subtracted from the values measured for each experimental condition, and the signal obtained with 10 mM Threonine was set as 100%. The mean and standard error values of three biological replicates are reported for each treatment. Asterisks in **(C)** indicate statistical significance difference between data of treatments and data of sterile water as determined by the Student’s *t*-test (^∗^*P <* 0.05, ^∗∗^*P <* 0.01). The experiment was carried out three times and one representative experiment is reported.

In Figure 9, *B. cereus* AR156 showed more effective colonizing ability on tomato root surfaces when co-cultured with 0.1 mM, 1 mM fructose, 0.1 mM lactic acid, 0.01 mM D-Pinitol, 1 mM, 10 mM sucrose, and 0.1, 1, and 10 mM threonine. The detected GFP signal of *B. cereus* AR156 colonization was then quantified and is shown in Figure 9B,C.

## Discussion

The proposed involvement of *B. cereus* AR156-mediated plant root exudates in biocontrol is consistent with the known functions of root exudates in regulating interactions between plants and PGPR (Zhou et al., 2016). However, no recent study has addressed the effects of *B. cereus* AR156-mediated plant root exudates on BCAs or pathogens. One hypothesis is that PGPR may manipulate the secretion of functional compositions in plant root exudates and play a positive role in biocontrol against plant diseases. Thus, it was important to explore and examine possible functional compositions in *B. cereus* AR156-mediated plant root exudates. The potential capability of inducing effective components in plant root exudates indicates the intricate patterns that *B. cereus* AR156 employs during biocontrol and is consistent with a previously reported recruitment strategy of the plant root system (Berendsen et al., 2012).

Recent advances in plant-microbe interaction research revealed that plants can shape their rhizosphere microbiome, as evidenced by the fact that different plant species host specific microbial communities when grown on the same soil (Berendsen et al., 2012). A study on root exudates of *Medicago truncatula* found that 15–20 compounds stimulated or repressed responses in QS (quorum sensing) reporter bacteria (Gao et al., 2003). In our study, the observation that compounds of tomato root exudates regulated the growth increment of *B. cereus* AR156 parallels results from other studies on pea (*Pisum sativum*), rice (*Oryza sativum*), and green algae (*Chlamydomonas reinhardtii*) (Teplitski et al., 2000, 2004; Ferluga and Venturi, 2009).

In GC-MS analysis, some of the components detected occurred in relatively low levels in root exudates from *B. cereus* AR156-treated tomato plants compared with non-treated groups, such as xylitol, myo-inositol, gamma-aminobutyric acid, and gluconic acid (Supplementary Table S1). Specifically, there was no signal detected for xylitol in all plants treated with *B. cereus* AR156. Xylitol is known as a sugar alcohol naturally found in plants that is used as an artificial sweetener in many foods, and it showed statistically significant inhibition at approximately 1.56% to *Streptococcus* strains (Sahni et al., 2002). It would be interesting to investigate the reasons for the low contents of these components in root exudates from *B. cereus* AR156-treated tomato plants. Additionally, a preliminary GC-MS analysis was conducted in the axenic culture of *B. cereus* AR156, in which out of six tested components in this work (fructose, lactic acid, D-pinitol, sucrose, threonine, and hexanoic acid), fructose, lactic acid, sucrose and threonine were detected (Supplementary Table S2). Although axenic culture of *B. cereus* AR156 was not the key objective of this study, it is interesting to understand the functions of *B. cereus* AR156 secretions during interactions between plant roots and rhizosphere bacteria.

Although specific components in REB showed positive effects on the growth and motile ability of *B. cereus* AR156, the concentration effects in this study should not be neglected. For example, only 10 mM hexanoic acid showed significant inhibitory effect on relative growth rate of *R. solanacearum* HN4, and the concentrations of hexanoic acid were 0.12 mM in REB, which partly explained the non-significant effects of crude root exudates on *R. solanacearum* HN4. Generally, if all tested metabolites were considered in the concentrations between 0.1 mM and 1 mM, it would be easier to understand the positive effects of REB on relative growth rate, swarming ability and biofilm forming ability of *B. cereus* AR156, and the non-significant effects of REW on *R. solanacearum* HN4 in all related results.

The concentration effects also exist in the antagonism assays presented in this study: although high concentrations of lactic acid and hexanoic acid had showed a bit antagonism activity in artificial condition, REB itself performed no detectable antagonism ability against *R. solanacearum* HN4, and the physiological concentrations of these tested metabolites in REB were far less than 50 and 100 mM. According to previous researches, 10 mM D-pinitol severely damaged conidiophores of *Podosphaera xanthii* which caused powdery mildew in cucumber; 12 mM hexanoic acid inhibited *in vitro* mycelial growth of germinated spores of *Botrytis cinerea*; 20 mM hexanoic acid effectively reduced *in vitro* infection and bacterial population development of *Xanthomonas citri* subsp. *ciri* (Leyva et al., 2010; Chen et al., 2014; Llorens et al., 2015), confirmed that REB plays no functional role in biocontrol against tomato bacterial wilt by directly affecting *R. solanacearum*, which was probably due to the low concentrations of effective metabolites in REB.

A study on biocontrol against root-knot nematodes revealed that a low concentration of lactic acid (0.3 mM) significantly inhibited the egg hatching rate of *M. incognita* (Lee et al., 2014). *B. cereus* AR156 was previously reported as an effective BCA strain for biocontrol against root-knot nematodes in tomato as well (Xu et al., 2017), which was somewhat consistent with the induction of lactic acid secretion in *B. cereus* AR156-treated tomato plants.

Interestingly, although 10 mM lactic acid and hexanoic acid showed no *in vitro* antagonism effect against *R. solanacearum* HN4, these two treatments still showed positive biocontrol efficacies against tomato bacterial wilt in greenhouse assays. In fact, hexanoic acid was previously reported to protect plants against *B. cinerea* by priming defense responses and reducing oxidative stress (Kravchuk et al., 2011; Ivan et al., 2014). These reports suggest that, despite of direct inhibition against the growth of pathogens, plant root exudates may be involved in *B. cereus* AR156-mediated biocontrol against other plant diseases by more complicated patterns, such as inducing ISR.

In a previous study, the motile ability of bacterial strains was proven to be associated with their biocontrol efficacy against plant diseases or related pathogenicity (Andersen et al., 2003; Wang et al., 2017). According to our research, only 0.72 mM lactic acid contributes to REB’s effects on the *in vitro* growth of *B. cereus* AR156, motile ability inhibition of *R. solanacearum* HN4, but not the growth rate of *R. solanacearum* HN4; 0.12 mM hexanoic acid in REB positively influenced *in vitro* growth of *B. cereus* AR156 and *R. solanacearum* HN4, the motile ability of *B. cereus* AR156, but not motile ability of *R. solanacearum* HN4, which partly explained the positive effect of REB in regulating the motile ability of *B. cereus* AR156, and indicated an other potential pattern REB takes in biocontrol process.

The biofilm formation ability of *B. subtilis* is known to play an important role in colonizing tomato roots and biocontrol against tomato disease caused by *R. solanacearum* (Chen et al., 2013). It was interesting to see if these conclusions could expand to other *Bacillus* species. In our data, 0.01 mM threonine induced the most robust *in vitro* biofilm formation of *B. cereus* AR156, while 10 mM threonine showed the most positive effect on the colonizing ability of *B. cereus* AR156 on the tomato root surface, indicating REB probably was more efficient in stimulating biofilm formation than root colonizing of *B. cereus* AR156 in *in vitro* condition. In addition, fructose, lactic acid, D-Pinitol, sucrose, and threonine were not exactly in their most effective concentrations in REB compared with the results of biofilm formation and colonizing ability assays. Considering these observations of root colonization of *B. cereus* AR156 occurred merely during the initial stage (3 days) and not in the soil environment, our future study will extend the detection period and repeat the root colonizing assay using more common and complex conditions.

The presented results in Figure 4, 5, 8, 9 demonstrated the effects of REB on the growth, swarming ability of *B. cereus* AR156 suggesting that the functional components in REB mainly affect the root colonization of *B. cereus* AR156 by ecological patterns, and these effects might also contribute to recruiting beneficial *Bacillus* spp. in root-soil system as previous reported (Berendsen et al., 2012). Meanwhile, although specific metabolites in high concentrations somewhat showed inhibitory effects, REB did not significantly affect the growth or swarming ability of *R. solanacearum* HN4, indicating a scarce relevance of REB on biocontrol aspects.

Collectively, the regulation of the growth of *B. cereus* AR156 by REB and the involvement of different plant root exudate components in the biocontrol process against tomato bacterial wilt suggest the important role of root exudates during the biocontrol process mediated by *B. cereus* AR156. This finding was consistent with previous research by Liu et al., who reported that *B. amyloliquefaciens* SQR9 induces the secretion of tryptophan in cucumber and that tryptophan further strengthens the colonization of SQR9 in cucumber roots (Liu et al., 2017). Indeed, some specific components in root exudates such as lactic acid and hexanoic acid displayed positive biocontrol efficacy against plant disease caused by *R. solanacearum* in greenhouse conditions. There were tremendous differences in the effects of root exudate components on biofilm formation and tomato root colonization of *B. cereus* AR156.

This study indicates the extended effects of REB on *B. cereus* AR156; however, our data were based on artificial environmental conditions, which have limitations to application in more complex and natural situations. For example, the colonizing test of *B. cereus* AR156 was in a pure culture system instead of soil. In the future, it would be particularly interesting to test our results in a natural environment and explore whether it could increase biocontrol efficacy by adding exogenous functional root exudate components to BCA products.

## Author Contributions

NW, LW, CJ, YQ, and J-HG designed the whole study. NW, LW, KZ, SH, LC, DM, and YG performed the experiments. NW, LW, CJ, YQ, and J-HG analyzed the data. LW, NW, YQ, CJ, and J-HG wrote the manuscript.

## Conflict of Interest Statement

The authors declare that the research was conducted in the absence of any commercial or financial relationships that could be construed as a potential conflict of interest.

## Supplementary Material

FIGURE S1 Metabolic concentrations of six induced metabolites in root exudates induced by *Bacillus cereus* AR156. Numbers above columns indicate fold change of each metabolite in tomato root exudate induced by *Bacillus cereus* AR156 (1 × 10^7^ CFU/mL) (REB) and tomato root exudate induced by sterile water (REW). Concentrations of each metabolite in REB or REW were calculated by comparison of analytes with standard sample as described by previous report (Pätzold et al., 2005). The mean and standard error values of six biological replicates are reported for each treatment. The experiment was carried out three times and one representative experiment is reported.

FIGURE S2 Antagonistic effects of components in tomato root exudates induced by *Bacillus cereus* AR156 against *Ralstonia solanacearum* HN4. **(A)** *In vitro* antagonism test of components in tomato root exudates induced by *B. cereus* AR156 against *R. solanacearum* HN4. The results in **(A)** are quantified in **(B)**. The mean and standard error values of three biological replicates are reported for each treatment. Different letters within a column in **(B)** indicate significantly differences among treatments by the Duncan’s multiple range tests test (*P <* 0.05). The experiment was carried out three times and one representative experiment is reported.

TABLE S1 GC-MS analysis data of root exudate from tomato root treated or non-treated with *B. cereus* AR156. Data is normalized by the internal standard L-norleucine within sample. The experiment was carried out three times and one representative experiment is reported.

TABLE S2 GC-MS analysis data of culture supernatant of *B. cereus* AR156. Data is normalized by the internal standard L-norleucine within sample. The experiment was carried out three times and one representative experiment is reported.

## References

[B1] Andersen J. B. Koch B. Nielsen T. H. Sørensen D. Hansen M. Nybroe O. (2003). Surface motility in *Pseudomonas* sp. DSS73 is required for efficient biological containment of the root-pathogenic microfungi *Rhizoctonia solani* and *Pythium ultimum*. *Microbiology* 149 37–46. 10.1099/mic.0.25859-0 12576578

[B2] Badri D. V. Loyola-Vargas V. M. Broeckling C. D. De-la-Peña C. Jasinaski M. Santelia D. (2008). Altered profile of secondary metabolites in the root exudates of *Arabidopsis* ATP-binding cassette transporter mutants. *Plant Physiol.* 146 762–771. 10.1104/pp.107.109587 18065561 PMC2245854

[B3] Badri D. V. Quintana N. Kassis E. G. E. Kim H. K. Choi Y. H. Sugiyama A. (2009). An ABC transporter mutation alters root exudation of phytochemicals that provoke an overhaul of natural soil microbiota. *Plant Physiol.* 151 2006–2017. 10.1104/pp.109.147462 19854857 PMC2785968

[B4] Barak J. D. Schroeder B. K. (2012). Interrelationships of food safety and plant pathology: the life cycle of human pathogens on plants. *Annu. Rev. Phytopathol.* 50 241–266. 10.1146/annurev-phyto-081211-172936 22656644

[B5] Berendsen R. L. Pieterse C. M. Bakker P. A. (2012). The rhizosphere microbiome and plant health. *Trends Plant Sci.* 17 478–486. 10.1016/j.tplants.2012.04.001 22564542

[B6] Bertin C. Yang X. Weston L. A. (2003). The role of root exudates and allelochemicals in the rhizosphere. *Plant Soil* 256 67–83. 10.1023/A:1026290508166

[B7] Bowsher A. W. Ali R. Harding S. A. Tsai C. J. Donovan L. A. (2015). Analysis of wild sunflower (*Helianthus annuus* L.) root exudates using gas chromatography-mass spectrometry. *J. Plant Nutr. Soil Sci.* 178 776–786. 10.1002/jpln.201400521

[B8] Chaparro J. M. Badri D. V. Bakker M. G. Sugiyama A. Manter D. K. Vivanco J. M. (2013). Root exudation of phytochemicals in *Arabidopsis* follows specific patterns that are developmentally programmed and correlate with soil microbial functions. *PLoS One* 8:e55731. 10.1371/annotation/51142aed-2d94-4195-8a8a-9cb24b3c733b 23383346 PMC3562227

[B9] Chen J. Fernandez D. Wang D. D. Chen Y. J. Dai G. H. (2014). Biological control mechanisms of D-pinitol against powdery mildew in cucumber. *Physiol. Mol. Plant Pathol.* 88 52–60. 10.1016/j.pmpp.2014.09.001

[B10] Chen Y. Cao S. G. Chai Y. R. Clardy J. Kolter R. Guo J. H. (2012). A *Bacillus subtilis* sensor kinase involved in triggering biofilm formation on the roots of tomato plants. *Mol. Microbiol.* 85 418–430. 10.1111/j.1365-2958.2012.08109.x 22716461 PMC3518419

[B11] Chen Y. Yan F. Chai Y. R. Liu H. X. Kolter R. Losick R. (2013). Biocontrol of tomato wilt disease by *Bacillus subtilis* isolates from natural environments depends on conserved genes mediating biofilm formation. *Environ. Microbiol.* 15 848–864. 10.1111/j.1462-2920.2012.02860.x 22934631 PMC3904073

[B12] Dakora F. D. Phillips D. A. (2002). Root exudates as mediators of mineral acquisition in low-nutrient environments. *Plant Soil* 245 35–47. 10.1023/A:1020809400075

[B13] Ferluga S. Venturi V. (2009). OryR is a LuxR-family protein involved in interkingdom signaling between pathogenic *Xanthomonas oryzae* pv. *oryzae* and rice. *J. Bacteriol.* 191 890–897. 10.1128/JB.01507-08 19028884 PMC2632065

[B14] Gao M. S. Teplitski M. Robinson J. B. Bauer W. D. (2003). Production of substances by *Medicago truncatula* that affect bacterial quorum sensing. *Mol. Plant Microbe Interact.* 16 827–834. 10.1094/MPMI.2003.16.9.827 12971606

[B15] Gao T. T. Foulston L. Chai Y. R. Wang Q. Losick R. (2015). Alternative modes of biofilm formation by plant-associated *Bacillus cereus*. *Microbiologyopen* 4 452–464. 10.1002/mbo3.251 25828975 PMC4475387

[B16] Huang J. F. Wei Z. Hu J. Yang C. L. Gu Y. A. Mei X. L. (2017). *Chryseobacterium nankingense* sp. nov. WR21 effectively suppresses *Ralstonia solanacearum* growth via intensive root exudates competition. *BioControl* 62 567–577. 10.1007/s10526-017-9812-1

[B17] Hückelhoven R. (2007). Transport and secretion in plant-microbe interactions. *Curr. Opin. Plant Biolol.* 10 573–579. 10.1016/j.pbi.2007.08.002 17875397

[B18] Ivan F. María D. L. O. L. P. Vicedo B. Rocío G. P. Jaime L. C. Pilar G. A. (2014). Hexanoic acid protects tomato plants against *Botrytis cinerea* by priming defence responses and reducing oxidative stress. *Mol. Plant Pathol.* 15 550–562. 10.1111/mpp.12112 24320938 PMC6638872

[B19] Jiang C. H. Fan Z. H. Xie P. Guo J. H. (2016). *Bacillus cereus* AR156 extracellular polysaccharides served as a novel micro-associated molecular pattern to induced systemic immunity to Pst DC3000 in *Arabidopsis*. *Front. Microbiol.* 7:664. 10.3389/fmicb.2016.00664 27242694 PMC4876362

[B20] Jin H. Qiao F. Chen L. Lu C. J. Xu L. Gao X. F. (2014). Serum metabolomic signatures of lymph node metastasis of esophageal squamous cell carcinoma. *J. Proteome Res.* 13 4091–4103. 10.1021/pr500483z 25162382

[B21] Kravchuk Z. Vicedo B. Flors V. Camañes G. González-Bosch C. García-Agustín P. (2011). Priming for JA-dependent defenses using hexanoic acid is an effective mechanism to protect *Arabidopsis* against *B. cinerea*. *J. Plant Physiol.* 168 359–366. 10.1016/j.jplph.2010.07.028 20950893

[B22] Lee Y. S. Naning K. W. Nguyen X. H. Kim S. B. Moon J. H. Kim K. Y. (2014). Ovicidal activity of lactic acid produced by *Lysobacter capsici* YS1215 on eggs of root-knot nematode, *Meloidogyne incognita*. *J. Microbiol. Biotechnol.* 24 1510–1515. 10.4014/jmb.1405.05014 25085571

[B23] Leyva M. O. Vicedo B. Finiti I. Flors V. Amo G. D. Real M. D. (2010). Preventive and post-infection control of *Botrytis cinerea* in tomato plants by hexanoic acid. *Plant Pathol.* 57 1038–1046. 10.1111/j.1365-3059.2008.01891.x

[B24] Liu Y. P. Chen L. Wu G. W. Feng H. C. Zhang G. Shen Q. R. (2017). Identification of root-secreted compounds involved in the communication between cucumber, the beneficial *Bacillus amyloliquefaciens*, and the soil-borne pathogen *Fusarium oxysporum*. *Mol. Plant Microbe Interact.* 30 53–62. 10.1094/MPMI-07-16-0131-R 27937752

[B25] Llorens E. Vicedo B. López M. M. Lapeña L. Graham J. H. García-Agustín P. (2015). Induced resistance in sweet orange against *Xanthomonas citri* subsp. *citri* by hexanoic acid. *Crop Protect.* 74 77–84. 10.1016/j.cropro.2015.04.008

[B26] Loyola-Vargas V. M. Broeckling C. D. Badri D. Vivanco J. M. (2007). Effect of transporters on the secretion of phytochemicals by the roots of *Arabidopsis thaliana*. *Planta* 225 301–310. 10.1007/s00425-006-0349-2 16868775

[B27] Narula N. Kothe E. Behl R. K. (2009). Role of root exudates in plant-microbe interactions. *J. Appl. Bot. Food Qual.* 82 122–130. 10.1614/IPSM-08-126.1

[B28] Nie P. P. Li X. Wang S. Guo J. H. Zhao H. W. Niu D. D. (2017). Induced systemic resistance against *Botrytis cinerea* by *Bacillus cereus* AR156 through a JA/ET- and NPR1-dependent signaling pathway and activates PAMP-triggered immunity in *Arabidopsis*. *Front. Plant Sci.* 8:238. 10.3389/fpls.2017.00238 28293243 PMC5329000

[B29] Niu D. D. Liu H. X. Jiang C. H. Wang Y. P. Wang Q. Y. Jin H. L. (2011). The plant growth promoting rhizobacterium *Bacillus cereus* AR156 induces systemic resistance in *Arabidopsis thaliana* by simultaneously activating salicylate- and jasmonate/ethylene dependent signaling pathway. *Mol. Plant Microbe Interact.* 24 533–542. 10.1094/MPMI-09-10-0213 21198361

[B30] Niu D. D. Wang C. J. Guo Y. H. Jiang C. H. Zhang W. Z. Wang Y. P. (2012). The plant growth-promoting rhizobacterium *Bacillus cereus* AR156 induces resistance in tomato with induction and priming of defence response. *Biocontrol Sci. Technol.* 22 991–1004. 10.1080/09583157.2012.706595

[B31] Niu D. D. Wang X. J. Wang Y. R. Song X. O. Wang J. S. Guo J. H. (2016a). *Bacillus cereus* AR156 activates PAMP-triggered immunity and induces a systemic acquired resistance through a NPR1-and SA-dependent signaling pathway. *Biochem. Biophys. Res. Commun.* 469 120–125. 10.1016/j.bbrc.2015.11.081 26616055

[B32] Niu D. D. Xia J. Jiang C. H. Qi B. B. Ling X. Y. Lin S. Y. (2016b). *Bacillus cereus* AR156 primes induced systemic resistance by suppressing miR825/825^∗^ and activating defense-related genes in *Arabidopsis*. *J. Integr. Plant Biol.* 58 426–439. 10.1111/jipb.12446 26526683 PMC5028193

[B33] Pätzold R. Schieber A. Brückner H. (2005). Gas chromatographic quantification of free D-amino acids in higher vertebrates. *Biomed. Chromatogr.* 19 466–473. 10.1002/bmc.515 16037932

[B34] Sahni P. S. Gillespie M. J. Botto R. W. Otsuka A. S. (2002). In vitro testing of xylitol as an anticariogenic agent. *Gen. Dent.* 50 340–343. 12640850

[B35] Shemesh M. Chai Y. R. (2013). A combination of glycerol and manganese promotes biofilm formation in *Bacillus subtilis* via histidine kinase KinD signaling. *J. Bacteriol.* 195 2747–2754. 10.1002/mbo3.251 23564171 PMC3697245

[B36] Sugiyama A. Shitan N. Yazaki K. (2007). Involvement of a soybean ATP-Binding cassette-type transporter in the secretion of genistein, a signal flavonoid in Legume-*Rhizobium* symbiosis. *Plant Physiol.* 144 2000–2008. 10.1104/pp.107.096727 17556512 PMC1949875

[B37] Suzuki K. Okazaki K. Tawaraya K. Osaki M. Shinano T. (2009). GC-MS associated global analysis of rice root exudates under aseptical condition. *Soil Sci. Plant Nutr.* 55 505–513. 10.1111/j.1747-0765.2009.00390.x

[B38] Teplitski M. Chen H. Rajamani S. Gao M. Merighi M. Sayre R. T. (2004). *Chlamydomonas reinhardtii* secretes compounds that mimic bacterial signals and interfere with quorum sensing regulation in bacteria. *Plant Physiol.* 134 137–146. 10.1104/pp.103.029918 14671013 PMC316294

[B39] Teplitski M. Robinson J. B. Bauer W. D. (2000). Plants secrete substances that mimic bacterial N-acyl homoserine lactone signal activities and affect population density-dependent behaviors in associated bacteria. *Mol. Plant Microbe Interact.* 13 637–648. 10.1094/MPMI.2000.13.6.637 10830263

[B40] Wang L. Y. Wang N. Mi D. D. Luo Y. M. Guo J. H. (2017). Poly-γ-glutamic acid productivity of *Bacillus subtilis* BsE1 has positive function in motility and biocontrol against *Fusarium graminearum*. *J. Microbiol.* 55 554–560. 10.1007/s12275-017-6589-y 28664519

[B41] Wang X. L. Wang L. Wang J. Jin P. Liu H. X. Zheng Y. H. (2014). *Bacillus cereus* AR156-induced resistance to *Colletotrichum acutatum* is associated with priming of defense responses in loquat fruit. *PLoS One* 9:e112494. 10.1371/journal.pone.0112494 25386680 PMC4227702

[B42] Wang X. L. Xu F. Wang J. Jin P. Zheng Y. H. (2013). *Bacillus cereus* AR156 induces resistance against *Rhizopus* rot through priming of defense responses in peach fruit. *Food Chem.* 136 400–406. 10.1016/j.foodchem.2012.09.032 23122077

[B43] Wei L. H. Xue Q. Y. Wei B. Q. Wang Y. M. Li S. M. Chen L. F. (2010). Screening of antagonistic bacterial strains against *Meloidogyne incognita* using protease activity. *Biocontrol Sci. Technol.* 20 739–750. 10.1080/09583151003714109

[B44] Wu K. Su L. Fang Z. Y. Yuan S. F. Wang L. L. Shen B. (2017). Competitive use of root exudates by *Bacillus amyloliquefaciens* with *Ralstonia solanacearum* decreases the pathogenic population density and effectively controls tomato bacterial wilt. *Sci. Hortic.* 218 132–138. 10.1016/j.scienta.2017.01.047

[B45] Xu S. Yang N. Zheng S. Y. Yan F. Jiang C. H. Yu Y. Y. (2017). The spo0A-sinI-sinR regulatory circuit plays an essential role in biofilm formation, nematicidal activities, and plant protection in *Bacillus cereus* AR156. *Mol. Plant Microbe Interact.* 30 603–619. 10.1094/MPMI-02-17-0042-R 28430084

[B46] Xue Q. Y. Yu C. Li S. M. Chen L. F. Ding G. C. Guo D. W. (2009). Evaluation of the strains of *Acinetobacter* and *Enterobacter* as potential biocontrol agents against *Ralstonia* wilt of tomato. *Biol. Control* 48 252–258. 10.1016/j.biocontrol.2008.11.004

[B47] Yan F. Yu Y. Y. Gozzi K. Yun C. Guo J. H. Chai Y. R. (2017). Genome-wide investigation of biofilm formation in *Bacillus cereus*. *Appl. Environ. Microbiol.* 83 e561–e617. 10.1128/AEM.00561-17 28432092 PMC5478996

[B48] Yazaki K. (2006). ABC transporters involved in the transport of plant secondary metabolites. *FEBS Lett.* 580 1183–1191. 10.1016/j.febslet.2005.12.009 16364309

[B49] Zhang N. Wang D. D. Liu Y. P. Li S. Q. Shen Q. R. Zhang R. F. (2014). Effects of different plant root exudates and their organic acid components on chemotaxis, biofilm formation and colonization by beneficial rhizosphere-associated bacterial strains. *Plant Soil* 374 689–700. 10.1007/s11104-013-1915-6

[B50] Zhou D. M. Huang X. F. Chaparro J. M. Badri D. V. Manter D. K. Vivanco J. M. (2016). Root and bacterial secretions regulate the interaction between plants and PGPR leading to distinct plant growth promotion effects. *Plant Soil* 401 259–272. 10.1007/s11104-015-2743-7

