# Supplementary figures and images for: Plant Root Exudates Are Involved in Bacillus cereus AR156 Mediated Biocontrol Against Ralstonia solanacearum
Source: Front Microbiol. 2019 Jan 31;10:98. doi: 10.3389/fmicb.2019.00098 (PMC6365458; doi:10.3389/fmicb.2019.00098)

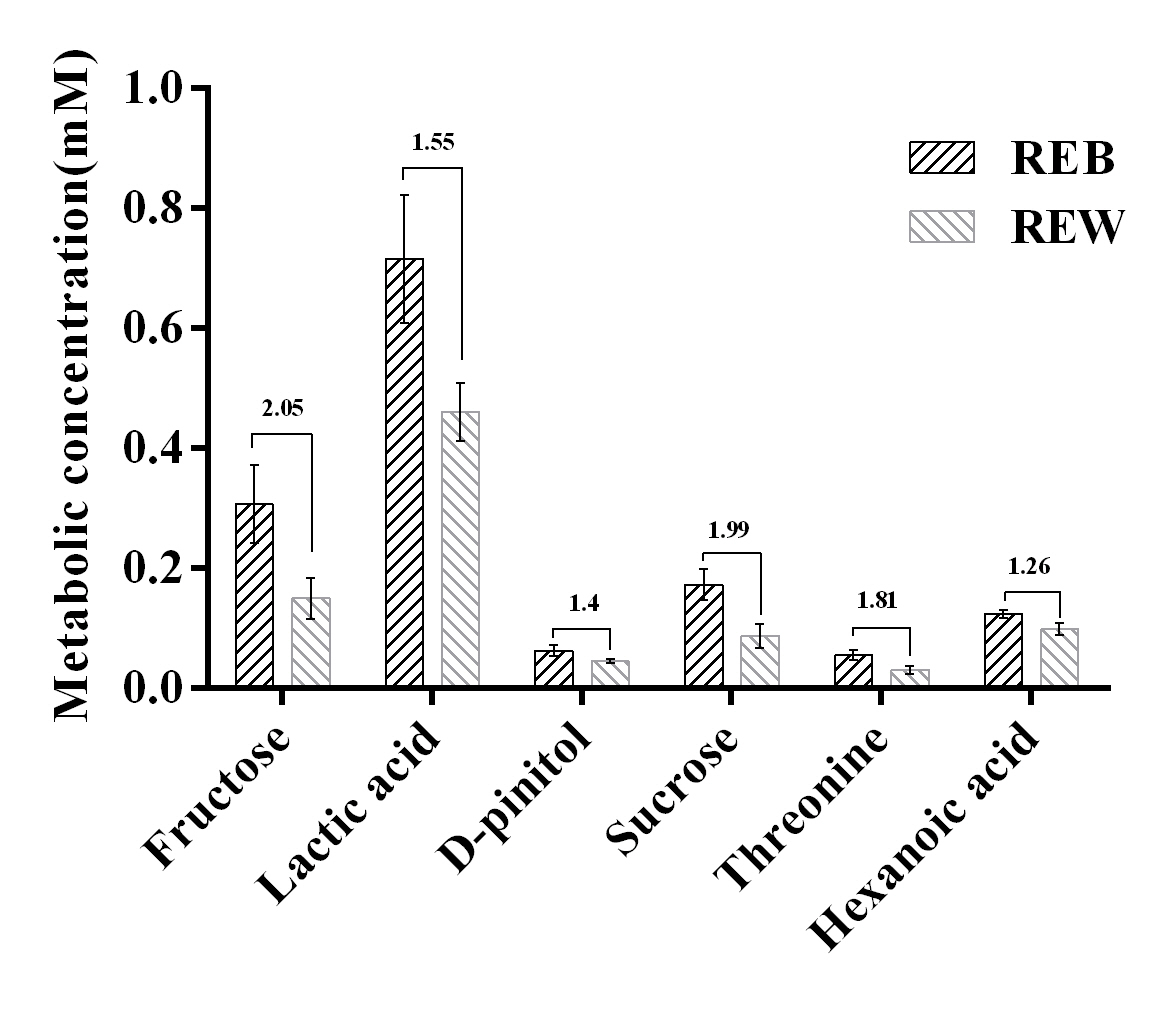

Supplement: FIGURE S1 — Metabolic concentrations of six induced metabolites in root exudates induced by Bacillus cereus AR156. Numbers above columns indicate fold change of each metabolite in tomato root exudate induced by Bacillus cereus AR156 (1 × 10^7^ CFU/mL) (REB) and tomato root exudate induced by sterile water (REW). Concentrations of each metabolite in REB or REW were calculated by comparison of analytes with standard sample as described by previous report (Pätzold et al., 2005). The mean and standard error values of six biological replicates are reported for each treatment. The experiment was carried out three times and one representative experiment is reported. [file Image_1.JPEG]

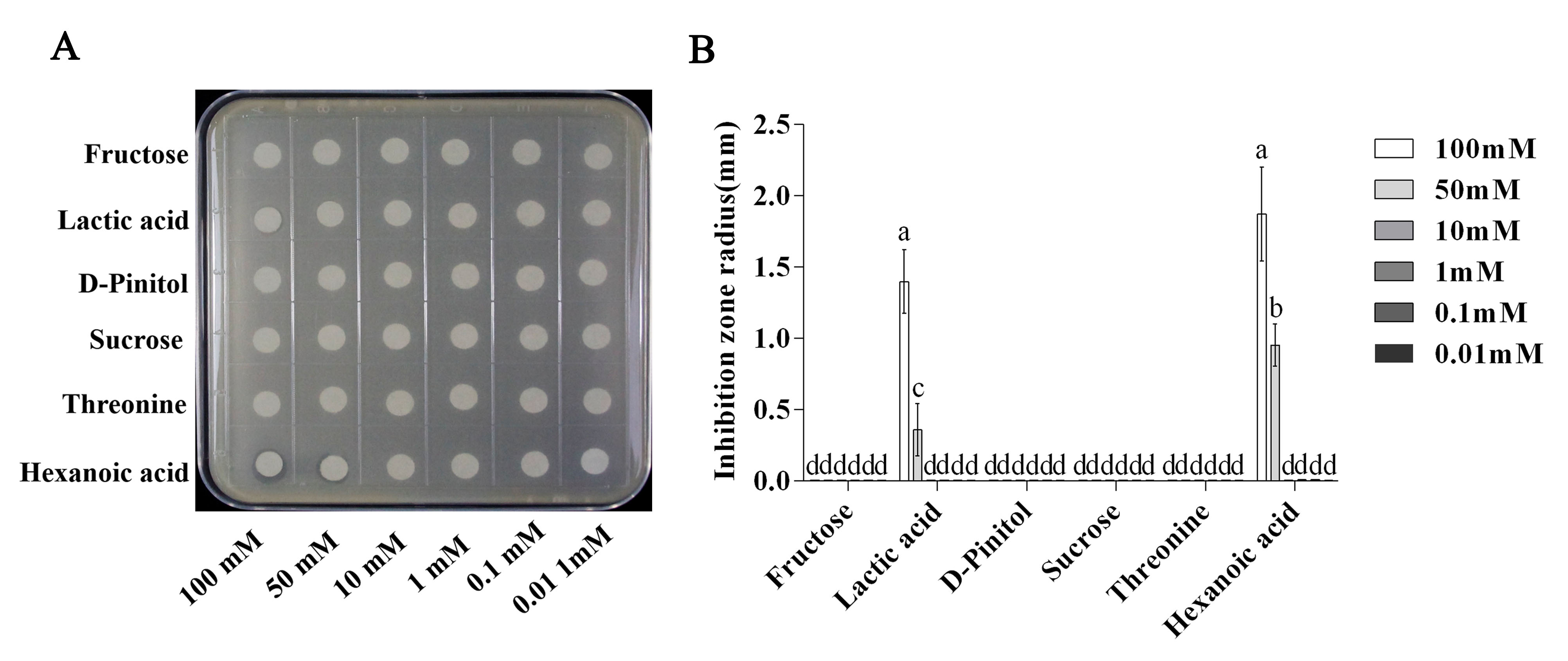

Supplement: FIGURE S2 — Antagonistic effects of components in tomato root exudates induced by Bacillus cereus AR156 against Ralstonia solanacearum HN4. (A) In vitro antagonism test of components in tomato root exudates induced by B. cereus AR156 against R. solanacearum HN4. The results in (A) are quantified in (B). The mean and standard error values of three biological replicates are reported for each treatment. Different letters within a column in (B) indicate significantly differences among treatments by the Duncan’s multiple range tests test (P < 0.05). The experiment was carried out three times and one representative experiment is reported. [file Image_2.JPEG]
